# Maternal and child health care access to skilled delivery services among Ghanaian rural mothers

**DOI:** 10.1007/s43999-024-00042-0

**Published:** 2024-04-24

**Authors:** Awinaba Amoah Adongo, Jonathan Mensah Dapaah, Francess Dufie Azumah, John Nachinaab Onzaberigu

**Affiliations:** Department of Sociology and Social Work Kwame, Nkrumah University of Science and Technology, Kumasi, Ghana

**Keywords:** Primary healthcare, Reproductive health needs, Maternal and child health, Community health. Rural areas

## Abstract

**Introduction:**

Most new-born babies are born at home in rural communities which is not new phenomenon due to lack of access to primary healthcare services and trained skilled health attendants, exposing mothers and children to a high risk of labour complications. The purpose of this study was to better understand factors influence rural women's access to primary health care and skilled delivery services as well as their reasons for using or not using maternal health care and skilled delivery services.

**Methods:**

The study employed a social survey design with a quantitative approach to data analysis. Cluster Sampling was used, possibly based on rural communities, to efficiently collect data from different geographic locations. Simple random sampling individuals from each cluster ensures that all eligible individuals have an equal chance of being included in the study. This enhances the representativity of the sample. A total of 366 mothers were selected from four rural communities in the North East Region of Ghana. The choice of sample size considered factors like the study's objectives, available resources, and the desired level of statistical power. Data was primarily gathered through the administration of a questionnaire to the respondents. Factors considered for achieving representativity include, geographic representation, accessibility, healthcare infrastructure and healthcare professionals’ attitudes**.**

**Findings:**

The study found that distance to health centres limits women's access to skilled delivery services. Lack of primary health facilities in the rural communities hamper maternal and child care services delivery. The attitude of health care professionals determines a mother’s utilisation of maternal health care and skilled delivery services.

**Conclusion:**

The study contributes to the limited research on maternal health services and their impact on mother and child health in the study area. This study is one of the first to investigate into maternal health care as a key predictor of mother and child health in the study area. The study's theoretical lens was the Andersen and Newman Health Behavioural Model theory, which supports the explanation of distance, lack of primary health centres, attitudes and lack of skilled personnel to the non-utilisation of maternal and health services in rural communities. The study recommended that primary healthcare facilities and trained health professionals should be a priority of government in rural communities to promote maternal and child healthcare.

## Introduction

Maternal and child health (MCH) is an international priority that has been debated for many years and is one of the most important public health service concerns (United Nations [UN], [1]). According to the United Nations, there were thousands of maternal deaths per year in 2019, and there were 18 infant fatalities per 100,000 live births worldwide. Sub-Saharan Africa and Southern Asia continue to record high maternal mortality rate globally (World Health Organisation [WHO], [2]). The Sustainable Development Goals (SDGs) set a target of a minimum of 70 maternal deaths per 100,000 babies born alive by 2030, however, maternal and child health issues continues to be an enormous obstacle in many countries. This suggests that the challenge of maternal and child still remains a concern to public particularly in sub-Saharan Africa.

The world's maternal mortality rate (MMR) in 2020 was 223 per 100,000 live births and to achieve a world-wide MMR of less than 70 by 2030 means that a yearly rate of reducing it drastically is required, an objective that has rarely been accomplished at the national level (WHO, [3]). Countries have banded together in the context of the SDGs to speeds up the decline in the death rate of mothers by 2030. SDG three includes the lofty objective of lowering the global MMR to less than 70 per 100,000 births, with none of the nation’s having an MMR that is more than multiple times the global mean. However, this is a major challenge in countries with limited resource setting, particular sub-Sharan Africa (SSA). There are numerous barriers to maternal and child health issues affecting maternal morbidity reduction in SSA. These factors could be attributed to poor health service location, a lack of health workers, a poor road network, and lack of service quality that put pregnant women at risk [4–6].

The MMR in Ghana is 310.00 per 100,000 live births, and the infant mortality rate is 31.78 deaths per 100,000 live births, according to Ghana Health Service (GHS, [7]). This an indication that the maternal and child mortality remains far short of the SDGs three target of 70 per 100,000 live births by 2030 in Ghana. The majority of maternal and infant mortality is triggered by the interaction of various economic, social, and health system factors such as lack of access to healthcare, health seeking behaviour, and socio-demographic behavioural variables [4, 5, 8, 9]. These variables require further evaluation and comprehension in order to address issues of maternal and infant mortality.

The government of Ghana's key policies for lowering maternal mortality rates include providing free maternal health care services and expanding healthcare facilities in rural communities (GHS, [7]). These policy interventions can reduce maternal mortality to some extent, the national MMR target of 80% in every 100,000 child births. However, it is unknown the situation of primary healthcare services and skilled trained health professionals in the rural context. Studies have shown that national indicators on women's access to and use of maternal health care services in Ghana, particularly urban areas, and little research focuses on maternal and child health in Ghana's rural communities (Awoonor-Willians, [10]; [11]). Furthermore, these factors are understudied and underappreciated in rural Ghana, where the majority of these maternal complications occur. As a result, the goal of this study was to understand maternal and child healthcare access to primary healthcare and skilled delivery services as well as the factors influencing their use of these in rural communities in Northern Ghana. Maternal health service quality has a greater positive effect on utilisation rates than service proximity. Also, the quality of maternal health care in hospitals were higher than in primary care facilities [12]. Similarly, the cost of medications may discourage mothers from seeking maternal healthcare services. Additionally, the availability of health personnel and maternal health policies were among the majority of reasons for mothers not using medications while pregnant; the review that follows provides empirical research on why mothers do or will not seek medications and health worker assistance when pregnant [13, 14]. 

There is gap in healthcare services in African countries and this often led to few people having access to healthcare facilities (WHO, [2]). As a result, money from developed countries has been the primary source of support for providing primary health care in many less developed countries worldwide (WHO, [15]). Donor funding for maternal health in less developed country has increased in recent years. Private clinics and hospitals are funded by entrepreneurs, so services from these health areas are prohibitively expensive for the average person to pay for pregnant women's health [16]. The improvement of health care centres and health professionals to provide maternal health service to mothers in rural areas, resulting in mothers' use of maternal health services in some rural areas where services are lacking, but most mothers in those areas may not seek maternal health service when they are pregnant because they may not have money or due to distance.

According to Iba et al. [17], requiring pregnant women to walk long distances and waste time before receiving health care and medication discourages many mothers. Similarly, if mothers do not have access to transportation to health care facilities that are far from their home, it will be difficult for them to access health care while pregnant because they will be unable to walk to the facility due to its location and how far it may be for the mother to go and get medication. There are a lot of literature on maternal health services that provide interesting findings, but there is a scarcity of research on maternal and child healthcare service access and skilled delivery services in rural communities in Ghana, so this study seeks to add to the literature.

### Study area and context

**Fig. 1:**
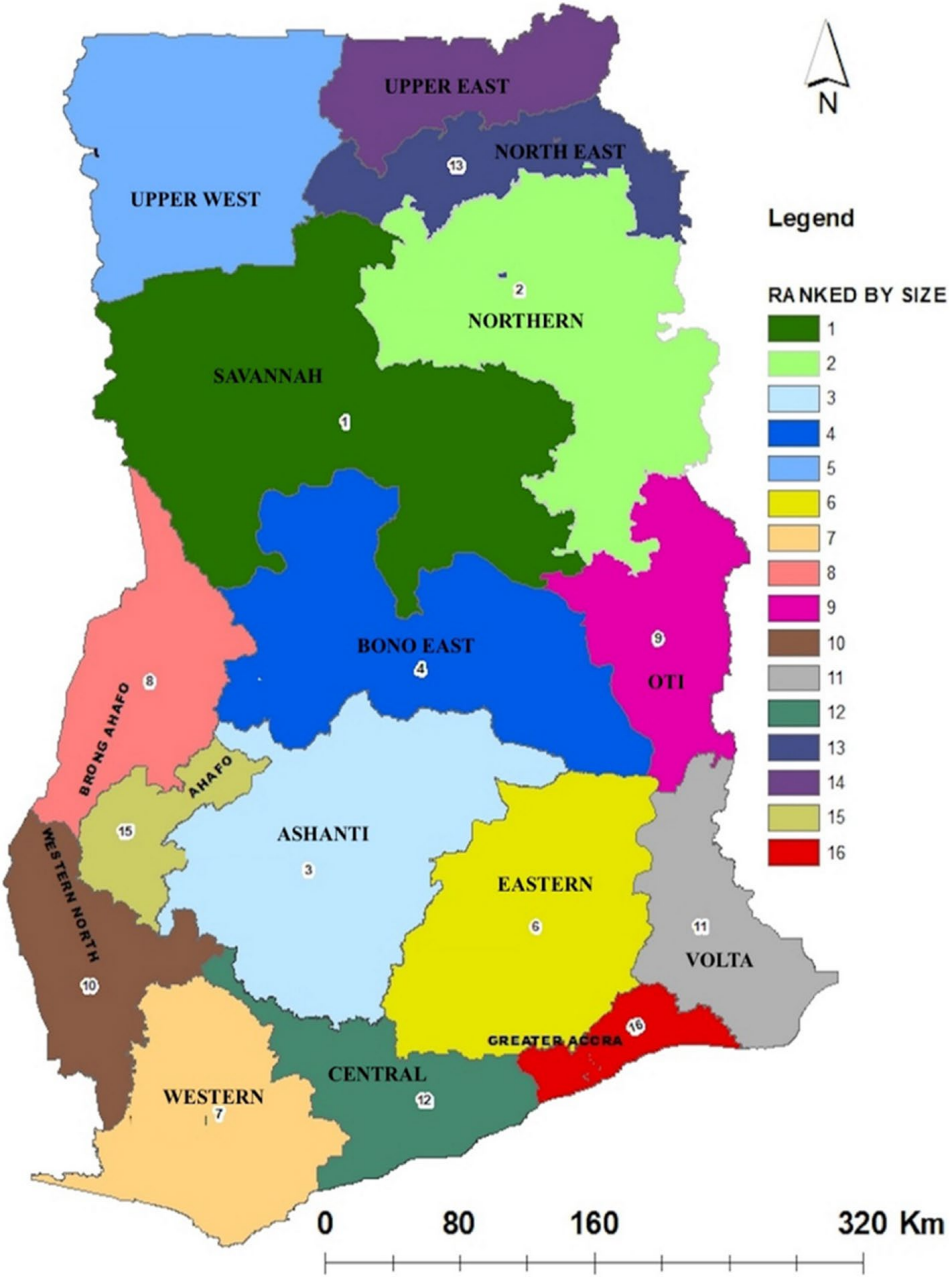
Map of Ghana, with the number 13 showing the study area

The study was conducted in four rural communities which included Yunyoo, Yagaba, Chereponi, and Bunkpurugu which are located in the North East Region of Ghana as indicated in Fig. 1. According to the GHS [7], there are the most rural communities in the North East Region of Ghana and lack primary healthcare services.

The study used a social survey design with probability techniques to sample mothers from four rural communities in Ghana's North East Region. The probability sampling method was used to select respondents from the larger population. It was necessary to ensure that everyone in the population has an equal chance of being selected for the survey, and that each respondent has an equal chance of being selected for the study, in order to ensure an accurate probability sample. Each person was assigned a number using a random number generator and the key was to avoid overrepresentation. The main benefit of probability sampling is that it allows the researcher to obtain accurate feedback about a larger population without polling the entire population. Simple random sampling was time consuming and tedious, especially with these larger samples [18].

Sampling in Ghana, like in many countries, is typically based on specific methodologies that aim to ensure representativeness of the population. However, achieving true random sampling can be challenging due to various constraints. Some concerns about potential limitations in achieving true random sampling include access and limited infrastructure and challenging terrain, especially in rural areas, can hinder the ability to reach and survey all segments of the population, leading to potential biases in the sample. Also, language barriers and cultural differences can affect the effectiveness of survey instruments and may result in underrepresentation. Unequal access to technology and digital devices may exclude certain segments of the population from surveys conducted through online or electronic means, introducing a potential bias in the sample. However, these limitations did not influence the results.

### Study population

The study population consists of women in fertility age (15–49 years) within four communities which included Yunyoo, Yagaba, Chereponi, and Bunkpurugu in the North East Region. It involved women who had ever become pregnant and were still at their reproductive age. The estimated population was 340,700 women (GHS, [7]). Women in fertility age 15–49 years were sampled from the four communities in the North East Region. There are approximately 340,700 women in the estimated population (GHS, [7]). The target population consisted of women aged 15 to 49 who had given birth within the previous five years. 

The sample size was derived with the aid of the formular below using a proportionate sample size at a confidence level of 95 per cent and confidence limit of 4. The confidence limit was reduced to 4 so as to get a larger sample size in order to have a more representative sample.

$$\mathbf{n}=\frac{{\varvec{D}}{\varvec{E}}{\varvec{F}}{\varvec{F}}.{\varvec{N}}{\varvec{p}}(1-{\varvec{P}})}{[\frac{{{\varvec{d}}}^{2}}{{{\varvec{Z}}}^{2\boldsymbol{ }\boldsymbol{ }}1-\boldsymbol{\alpha }|2}.\left({\varvec{N}}-1\right)+{\varvec{P}}\left(1-{\varvec{P}}\right)]}$$

where:

n: This represents the required sample size.

DEFF: Design Effect. It accounts for the design effect or the impact of clustering in the sampling process. If previous data has been collected in clusters or groups, the design effect adjusts the sample size to reflect the fact that observations within the same cluster may be more similar to each other than to observations in other clusters.

N: Total population size.

p: The estimated proportion of the population that has a certain characteristic. It is the proportion the researchers want to estimate.

1 − p: The complement of the estimated proportion.

d^2^: This represents the desired margin of error (precision) in estimating the population proportion.

Z_1_ − α/2: The Z-score corresponding to the desired level of confidence (1 − α). This is often denoted as the critical value and is associated with the confidence level for the estimation.

α: The significance level, which is typically set at 0.05 for a 95% confidence level.

How population is organised in Ghana include the National Identification Authority (NIA) which is the primary government agency responsible for the registration and issuance of national identification cards. They conduct mass registration exercises to capture the biometric data of citizens, including fingerprints and facial features. Also, the Birth and Death Registry is responsible for registering all births and deaths in the country. Birth registration is crucial for establishing citizenship and providing individuals with official identification. Ghana Statistical Service (GSS, [19]) also plays a role in population data collection through censuses and surveys. These initiatives help in obtaining demographic information, economic indicators, and other relevant data. The Electoral Commission manages voter registration, which is a subset of the overall population registration. While its primary focus is on citizens eligible to vote, it contributes to the overall demographic data ([20], GSS, [19]).

With regard to completeness of population registration, especially in rural areas achieving complete population registration in rural areas can be challenging due to factors such as limited infrastructure, poor accessibility, and lower awareness of the importance of registration. To address challenges in rural areas, mobile registration units are often deployed. These units travel to remote locations to ensure that residents, even in distant and less accessible areas, have the opportunity to register. The government and relevant agencies conduct public awareness campaigns to educate citizens about the importance of registration. These campaigns often target rural areas where awareness might be lower. Collaborating with local authorities, community leaders, and grassroots organisations is essential for ensuring that registration efforts are accepted and supported at the community level ([20],GSS, [19]).

## Methods

### Theoretical context: behavioural model by andersen and newman in 1973

The study used the Andersen and Newman Health Behavioural Model (1973) to identify the factors influence the utilisation of health facilities in the rural communities. Health Behavioural Model states that some feature influence mothers to look for health services when pregnant. These features are in three set that may influence mothers to use health service when pregnant arrive: (1) predispose factor; (2) Enabling resources, (3) Actual or perceived need.

### Predisposing characteristics

Factors which influence mothers to use MHS which may be the age at pregnancy, whether the early birth was successful, social factors, the work the mother does, the level the mother has be educated up to, the works the family do and whether the family has enough money [21]. When a mother has most of these factors, she is most likely to use services at health facility during pregnancy but if not, the mother will even find it difficult to afford health cost given a situation when the mother has no money or from a poor family.

### Enabling characteristics

With regards to enabling characteristics, the health behavioural model has it that some factors have to be available for mother to be able to use health service. These factors are termed as enabling since they make access to health service easy to mothers. There are within the community and area the mother lives. Family factors which may include their level of income, family doctor, health insurance policy that mother may be with, the distance of mother’s house and the any nearby health facility [21].

Families with their own doctors will imply that the mother can easily get treatment for health services when pregnant but those families without a family doctor will have to rely on the community doctors at the private or public health centre. The enabling factors are central to mothers seeking for health services during pregnancy**.**

The next factor is the need-based factor of mothers and them looking for health services when pregnant. When mothers think that there is the need for them to look for medications during pregnancy then they will look for medications, but when mothers do not foresee the need then they will not look for health when pregnant (Anderson & Newman, [22]; [23, 24]). The Andersen and Newman [25] model was used in this study to help identify the determinants of one's health service utilisation, assess inequality in access to health services, and facilitate policy-making for equitable access to care and health services in rural communities.

### SData collection, management and analysis techniques

SIn this study, the target population included mothers in rural communities within the North East Region of Ghana. Clusters were identified which were the rural communities. A random selection method was to choose clusters from the larger population of rural communities in the North East Region. Contacting participants was done through community engagement. The research established contact with community leaders and members to explain the purpose and importance of the study and seek their support and cooperation. Obtained informed consent from potential participants, ensuring they understand the study and voluntarily agree to participate. The authors used systematic sampling within the selected clusters to identify households or individuals. A structured questionnaire was used to collect quantitative data on factors influencing women's access to primary healthcare and skilled delivery services [18].

The train enumerators were PhD Candidates who administered the questionnaire. The candidates with training in both quantitative and qualitative research methodologies ensure a robust and nuanced approach to data collection. They were also familiar with the local culture and language to facilitate effective communication to assist participants who could not read. Ensuring inclusivity, the researchers conduct extensive community outreach to ensure that potential participants from diverse backgrounds and situations are aware of the study. Accessibility measures, the researchers implement measures to make the study accessible to all eligible participants, considering factors such as mobility, language barriers, and cultural sensitivity.

The dependent variables were utilisation of maternal health care and skilled delivery services. This variable was operationalised as a binary outcome – utilised or did not utilise maternal health care and skilled delivery services. Based on responses to survey questions regarding participants' utilisation of maternal health care services during pregnancy and delivery. The independent variables were distance to health centres. The distance in kilometres from participants' residences to the nearest health centres. Measured using GIS or self-reported estimates during interviews. Another independent variable was availability of primary health facilities and the presence or absence of primary health facilities in the participants' rural communities. The last independent variable was attitude of health care professionals and participants' perception of health care professionals' attitudes, measured through Likert scale questions in the survey and it was measured based on responses to questions assessing participants' satisfaction or dissatisfaction with the attitudes of health care professionals.

Additional information on previous pregnancies, births, and complications were captured data on the number of previous pregnancies, births, and any complications experienced during these events. Participants provided the number of previous pregnancies and births, and details on complications were recorded based on participants' self-reporting. Assess the impact of genital mutilation on maternal health care utilisation included questions in the survey that inquire about participants' experiences with genital mutilation and its potential influence on seeking maternal health care services (GHS,[7]; GSS, [19]).

Data cleaning process the authors identified and addressed missing data through imputation or exclusion, depending on the extent of missingness and patterns. Also, we use statistical methods to identify and investigate outliers that may skew the data. The authors adjusted or removed outliers based on the study's goals. Examined data for inconsistencies, ensuring that responses are logical and coherent. Addressed any conflicting information through cross-validation or follow-up with participants. Validated the coding of categorical variables and ensure consistency in coding schemes throughout the dataset. Checked for errors in data entry and correct any inaccuracies. Cross-verify with original survey instruments [26].

Quantitative analysis was used which involved descriptive statistics and inferential statistics. The quantitative approach was used to ensure that major variables were numerically measured and to provide statistical data on factors influencing women's use of maternal and skilled delivery care. The quantitative approach helped the researchers to gather empirical data that gave readers and policy makers a better understanding of mother’s use and non-use of maternal and skilled delivery care using quantifiable data [18].

## Results

### Respondents’ background information

According to the data in Table 1, 15.6% (*n* = 57) of the 366 respondents were single, while 63.1% (*n* = 231) of the respondents were in a marital union recognised by the community. Only 6.8% (*n* = 25) of the respondents had divorced, 10.9% (*n* = 40) had lost their partners (widow), and 3.6% (*n* = 13) were in a cohabitation relationship. Respondents between the ages of 31 and 35 account for the highest percentage of 28.1% (*n* = 103). This was followed by those aged 26–30 years, who had a percentage of 24% (*n* = 88). Respondents aged 46–49 years comprised the minority group, with a valid percentage of 4.4% (*n* = 16). Again, a close examination of the respondents' age range revealed that very few of those who participated in the study were aged years or less. According to the study, 47% of respondents (*n* = 172) had no formal education. At least 24% (*n* = 88) and 20.5% (*n* = 75) of respondents had only basic/primary education, respectively. The valid percentages for senior high school (including technical and vocational education) and tertiary education were 5.5% (*n* = 20) and 3% (*n* = 11), respectively. The majority of them 47.8% (*n* = 175) of respondents were traditional believers, 43.4% (*n* = 159) of respondents were Christians, 7.4% (*n* = 27) of respondents were Muslims, 1.4% (*n* = 5) of respondents were free thinkers, and 92% (*n* = 335) of respondents cannot read.

**Table 1:** Socio-demographic Characteristic of Respondents

Variable/ Categories	Frequency *n* = 366	Percent %
**Marital status**		
Single	57	15.6
Married	231	63.1
Divorced	25	6.8
Widow	40	10.9
Cohabitation relationship	13	3.6
**Age group**		
15–20 years	51	13.9
21–25 years	57	15.6
26–30 years	88	24.0
31–35 years	103	28.1
36–40 years	32	8.7
41–45 years	19	5.2
46–49 years	16	4.4
**Age at last birth (within past five years 2015–2020)**		
less than 20 years	62	16.9
21–25 years	64	17.5
26–30 years	140	38.3
31–35 years	75	20.5
36–40 years	15	4.1
41 years and above	10	2.7
**Level of education**		
No formal education	172	47.0
Basic/primary level	88	24.0
Junior high level	75	20.5
Senior high level	20	5.5
Tertiary level	11	3.0
Total number women who cannot read	335	92.0
**Religious affiliation**		
Christianity	159	43.4
Muslim	27	7.4
Traditionalist	175	47.8
Free thinkers	5	1.4

**Source: Author’s Field Work (2021)**

### Mothers’ place of delivery during child birth

The study established that more births by mothers being delivered at home than child births that occurred at the hospital/clinic, with the exception of most mothers having their third child at the hospital. Most mothers who had more than one child (two children) delivered at home 47% of the time, compared to 27.8% and 2.2% of mothers who delivered at the hospital and clinic, respectively. According to the study, only 37.7% (*n* = 138) of mothers had their third delivery in a hospital, while the majority of respondents (63.9%) had their last birth at home. According to field data, most women still deliver at home, according to a general examination of mothers' places of delivery in the North East Region. However, one cannot deny that the factor plays a significant role; 4 out of every 10 mothers had their first child at a hospital/clinic. 

### Month mothers received ANC during pregnancy

Table 2 shows the months when the majority of respondents visited the hospital during their previous pregnancy, according to the findings. The majority of mothers in the North East Region (37.2% and 38.3%, respectively) began visiting ANC during the second and third months of their pregnancy, according to data in Table 2. Only 7.1% of mothers sought ANC during the first month of their pregnancy. According to Table 2, 94.3% (*n* = 345) of respondents had a live birth within the last five years during their most recent childbirth. Only 5.7% (*n* = 21) of those polled experienced a stillbirth during their most recent pregnancy.

**Table 2:** Mothers maternal health care history

Variable/categories	Frequency *n* = 366	Percent %
**Month mothers received ANC during pregnancy**		
First month of pregnancy	26	7.1
Second month of pregnancy	136	37.2
Third month of pregnancy	140	38.3
Fourth month of pregnancy	50	13.7
Fifth month of pregnancy	6	1.6
Sixth month of pregnancy	3	.8
Can’t tell	5	1.4
**Outcome of last birth**		
Live birth	345	94.3
Still birth	21	5.7

**Source: Author’s Field Work (2021)**

### Univariate analyses (chi-square testing)

The researchers investigated the factors that influence women's use of maternal health care services in the North East Region, as well as access to skilled delivery services. The dependent variables were ANC use, delivery care at a health centre, and postnatal care at a health centre, while the independent variables were marital status, age, education, religion, distance, and the availability of skilled delivery personnel. The test results are shown in Tables 6, 7, and 8 using the Chi-square (2) and an alpha of 0.05 (= 0.05).

The Chi-square (***χ***^**2**^) was computed to determine whether socio-demographic factors such as marital status, age, education, religion, distance, and availability of skilled delivery personnel have a relationship with mothers' utilisation of ANC. The five married categories were recoded as single and married; age group was recoded as youth and older; education was recoded as not educated and educated; and distance was recoded as within the community and far from the community. This was done to ensure that each cell had at least five respondents, as the initial codes resulted in some cells having fewer than five, which was not consistent with the Chi-square test assumptions.

The Chi-square (***χ***^**2**^) test results as depicted in Table 3 indicated a statistically significant relationship between marital status ***χ***^**2**^(1, *n* = 366) = 6.281, *p*-value = 0.012(S), ά = 0.05, age of mothers ***χ***^**2**^ (1, *n* = 366) = 8.702, *p*-value = 0.003, education of mothers ***χ***^**2**^ (1, *n* = 366) = 4.007, *p*-value = 0.045, religion ***χ***^**2**^(2, *n* = 366) = 12.124, *p*-value 0.002, and distance ***χ***^**2**^ (1,*n* = 366) = 5.393, *p*-value = 0.020, all at ά = 0.05 and mothers’ utilisation of maternal health care services in the North East Region.

**Table 3:** Socio-demographic and Utilization of antenatal care service (ANC)

Variables						
		No	Yes	Total *N* = 366	**Chi-square *χ***^**2**^	***P*-value**
Marital status	Single	99(27.0%)	25(6.8%)	124(33.9%)	6.281	0.012
	Married	163(44.5%)	79(21.6%)	242(66.1%)		
Age	Youth < 30	153(41.8%)	43(11.7%)	196(53.6%)	8.702	0.003
	Older ˃30	109(29.8%)	61(16.7%)	170(46.4%)		
Education	Not educated	235(64.2%)	100(27.3%	335(91.5)	4.007	0.045
	Educated	27(7.4%)	4(1.1%)	31(8.5%)		
Religion	Christian	119(32.5%)	41(11.2%)	160(43.7%)	12.124	0.002
	Muslim	26(7.1%)	1(0.3%)	27(7.4%)		
	Traditional	117(32.0%)	62(16.9%)	179(48.9%)		
Distance	Within the community	82(22.4%)	20(5.5%)	102(27.9%)	5.393	0.020
	Far from the community	180(49.2%)	84(23.0%)	264(72.1%)		
Availability of skilled delivery personnel	Yes	214(58.5%)	82(22.4%)	296(80.9%)	0.386	0.534
	No	48(13.1%)	22(6.0%)	70(19.1%)		

Source: Author’s Field Work (2021)

The Chi-square ***χ***^**2**^ test results of the mothers' socio-demographic factors, such as married status, age, education, religious background, and distance, were found to have a significant relationship with their use of ANC. When mothers marry, their partners contribute to MHC costs such as transportation to the health care centre. Similarly, older women learn through experience whether they need to access MHCS or not, whereas educated young women have a better understanding of the need for maternal health care services during pregnancy. To determine whether mothers’ socio-demographic factors have relationship on utilisation of delivery care at the health, the Chi-square (***χ***^**2**^) was used as depicted in Table 4. The Chi-square (***χ***^**2**^) in Tables 4 and 5 showed that there is significant relationship between mothers’ marital status, ***χ***^**2**^ (1, *n* = 366) = 10.909, *p*-value = 0.001, age of mothers ***χ***^**2**^ (1, *n* = 366) = 5.222, *p*-value = 0.022, mothers’ education ***χ***^**2**^ (1, *n* = 366) = 7.893, *p*-value = 0.005, religious background ***χ***^**2**^ (2, *n* = 366) = 10.020, *p*-value = 0.007 and distance ***χ***^**2**^ (1, *n* = 366) = 7.372, *p*-value 0.007, at ά = 0.05.

**Table 4:** Socio-demographic and Utilization of delivery care at health centre

Variables						
		No	Yes	Total *N* = 366	**Chi-square *χ***^**2**^	***P*-value**
Marital status	Single	98(26.8%)	26(7.1%)	124(33.9%)	10.909	0.001
	Married	150(41.0%)	92(25.1%)	242(66.1%)		
Age	Youth < 30	143(39.1%)	53(14.5%)	196(53.6%)	5.222	0.022
	Older ˃30	105(28.7%)	65(17.8%)	170(46.4%)		
Education	Uneducated	220(60.1%)	115(31.4%)	335(91.5%)	7.893	0.005
	Educated	21(5.7%)	10(2.8%)	31(8.5%)		
Religion	Christian	111(30.3%)	49(13.4%)	160(43.7%)	10.020	0.007
	Muslim	21(5.7%)	6(1.7%)	27(7.4%)		
	Traditional	112(30.6%)	67(18.3%)	179(48.9%)		
Distance	Within the community	80(21.9%)	22(6.0%)	102(27.9%)	7.372	0.007
	Far from the community	168(45.9%	86(26.2%)	264(72.1%)		
Availability of skilled delivery personnel	Yes	202(55.2%)	94(25.7%)	296(80.9%)	0.166	0.684
	No	46(12.6%)	24(6.6%)	70(19.1%)		

**Source: Author’s Field Work (2021)**

**Table 5:** Socio-demographic and Utilization of Postnatal care at health centre

Variables						
		**No**	**Yes**	**Total N = 366**	**Chi-square *χ***^**2**^	***P*-value**
Marital status	Single	99(27.0%)	25(6.8%)	124(33.9%)	11.522	0.001
	Married	151(41.3%)	91(24.9%)	242(66.1%)		
Age	Youth < 30	140(38.3%)	56(15.3%)	196(53.6%)	1.901	0.168
	Older ˃30	110(30.1%)	60(16.4%)	170(46.4%)		
Education	Not educated	227(62.0%)	108(29.5%)	335(91.5%)	0.542	0.461
	Educated	23(6.3%)	8(2.2%)	31(8.5%)		
Religion	Christian	110(30.1%)	50(13.7%)	160(43.7%)	11.550	0.003
	Muslim	20(5.5%)	7(1.9%)	27(7.4%)		
	Traditional	114(31.1%)	65(17.8%)	179(48.9%)		
Distance	Within the community	76(20.8%)	26(7.1%)	102(27.9%)	2.514	0.113
	Far from the community	174(47.5%	90(24.6%)	264(72.1)		
Availability of skilled delivery personnel	Yes	203(55.5%)	93(25.4%)	296(80.9%)	0.054	0.816
	No	47(12.8%)	23(6.3%)	70(19.1%)		

**Source: Author’s Field Work (2021)**

### Logistic regression model

To identify socio-demographic factors that influence the likelihood that mothers in the North East Region would use maternal health care services, binary logistic regression was used. Six independent variables were included in the models (Marital status, Age, Education, Religion, Distance and Availability of skilled delivery personnel). Three different models were developed, each with antenatal care, delivery care at a health centre, and postnatal care as separate dependent variables. The first model, which included mothers’ socio-demographics and use of ANC, contained all statistically significant predictors, ***χ***^**2**^ (7, *n* = 366) = 29.649, *p*-value = 0.000, indicating that the model was able to distinguish between mothers who used and did not use ANC during pregnancy and which explained 7.8% (Cox and Snell R square) and 11.2% (Nagelkerke R square) of the variation in mothers' utilisation of antenatal care in the North.

The second model included mothers' socio-demographic information as well as their use of delivery care in the North East Region. This model was also statistically significant, ***χ***^**2**^ (7, *n* = 366) = 33.312, *p*-value = 0.000, implying that the second model could predict mothers who used delivery care at a health centre during childbirth and those who did not in the North East Region. This explained between 8.7% (Cox & Snell R Square) and 12.2% (Nagelkerke R Square) of the variations in mothers' use of delivery care in the district (see Table 7).

The last model was computed to determine mothers’ socio-demographic and use of postnatal care in health centre after child birth. This model of showed statistically significant, χ2 (7, *n* = 366) = 27.203, *p*-value = 0.000, meaning that it was able to predict mothers who used postnatal care at health centre after child birth and those who did not, which also explained between 7.2% (Cox & Snell R Square) and 10% (Nagelkerke R Square) of the variation in mothers use of postnatal care at health centre after child birth as showed in Table 8.

As showed in model 1 (Table 6) and model 2 (Table 7) only one independent variable (education) made a statistically significant contribution to the models which predicted mothers’ use of ANC and delivery care whilst in model 3 (Table 8) only one independent variable (availability of skilled delivery personnel) of mothers made statistically significant contribution to the model which predicted mothers’ use of postnatal care at health centre after child birth. An elaborated interpretation of the results with regards to the various statistics is presented beneath the Tables 6, 7 and 8.

**Table 6:** Binary Logistic regression predicting socio-demographic and use of antenatal care (*n* = 366)

Variables	Categories	B	Wald	*p*-value	Exp(B)
Marital status	Single	1			
	Married	-.460	2.837	.092	.631
Age	Youth < 30	1			
	Older > 30	-.511	4.375	.036	.600
Education	No educated	1			
	Educated	.607	1.119	.290	1.836
Religion	Christian	1			
	Non-Christian	-2.300	4.904	.027	.100
Distance	Within the community	1			
	Far from the community	-.452	2.329	.127	.636
Availability of skilled delivery personnel	No	1			
	Yes	-.003	.000	.993	.997
Constant		-.762	1.291	.256	.467

Cox & Snell R Square = 0.078

Nagelkerke R Square = 0.112

Chi-square (χ2) (7, *n* = 366) = 29.649

*p*-value = 0.000

**Source: Author’s Field Work (2021)**

**Table 7:** Binary Logistic regression predicting socio-demographic and use of skilled delivery care (*n* = 366)

Variables	Categories	B	Wald	*p*-value	Exp(B)
Marital status	Single	1			
	Married	-.672	6.345	.012	.511
Age	Youth < 30	1			
	Older > 30	-.308	1.692	.193	.735
Education	No educated	1			
	Educated	1.221	3.670	.055	3.390
Religion	Christian	1			
	Non-Christian	-1.685	4.877	.027	.185
Distance	Within the community	1			
	Far from the community	-.490	2.920	.087	.613
Availability of skilled delivery personnel	No	1			
	Yes	.075	.065	.799	1.078
Constant		-1.320	3.364	.067	.267

Cox & Snell R Square = 0.087

Nagelkerke R Square = 0.122

Chi-square (χ2) (7, *n* = 366) = 33.312

*p*-value = 0.000

**Source: Author’s Field Work (2021)**

**Table 8:** Binary Logistic regression predicting socio-demographic and use of postnatal care (*n* = 366)

Variables	Categories	B	Wald	*p*-value	Exp(B)
Marital status	Single	1			
	Married	-.811	9.073	.003	.444
Age	Youth < 30	1			
	Older > 30	-.106	.200	.654	.900
Education	No educated	1			
	Educated	-.061	.017	.895	.941
Religion	Christian	1			
	Non-Christian	-2.600	6.260	.012	.074
Distance	Within the community	1			
	Far from the community	-.214	.593	.441	.807
Availability of skilled delivery personnel	No	1			
	Yes	.028	.009	.925	1.028
Constant		-.206	.127	.721	.814

Cox & Snell R Square = 0.072

Nagelkerke R Square = 0.100

Chi-square (χ2) (7, *n* = 366) = 27.203

*p*-value = 0.000

**Source: Author’s Field Work (2021)**

The beta (B) values in the first column of Table 6 represented the co-efficient of the independent variables entered into the model. From the Table 6, it was observed that education of mothers recorded the highest beta value (0.607) and with the least value of beta recorded for religious (-2.300). This means that education is a strong predictor of utilisation of ANC in the North East Region. Only education influence use of ANC services positively.

The Wald values in the second column of Table 6 also indicated the contribution made by each of the independent variables to the model which predicts the utilisation of ANC services in the North East Region. As indicated Table 6, it was religion (4.904) and age (4.375) which mostly made significant contribution to mothers’ use of ANC services.

The *p*-values in the Table 6 also indicated the variable or set of variables that significantly predict mothers’ utilisation of ANC services in the North East Region. From the results in Table 6, only religion (0.035) and age (0.027) proved to be the only significant determinants or predictors of mothers’ utilisation (model) of ANC in the North East Region, as they recorded *p*-values less than 0.05. The last column in Table 6 indicated the odds ratios (Exp(B)) for each of the independent’s variables. To determine which socio-demographic factors were strong predictors of mothers’ use of postnatal care services in the North East Region. The beta (B) values in the beta (B) column of Table 8 showed that only NHIS (B = 0.028) influences mothers’ use of postnatal care positively in the North East Region. All the others predictors have negative influence on mothers’ use of postnatal care. The Wald column in Table 8 showed that marital status (9.073) and religion (6.260) made the most contribution to the model in predicting mothers’ socio-demographic factors and use of postnatal care services whilst education and availability of skilled delivery personnel made the less contribution to the model with Wald values of (0.017 and 0.009) respectively.

The model on mothers’ socio-demographic and use of delivery care showed that education recorded that the highest Beta (B) of (1.221) meaning that education is a strong predictor of mothers’ utilisation of delivery care positively. The data in Table 7 showed that religion negatively influence mothers’ use of delivery care than all the others variables. A critical looked at all the Beta (B) showed that all the variables except education and Availability of skilled delivery personnel influence mothers’ use of skilled delivery care negatively. The study further found that marital status with Wald value of (6.345) and religion with Wald value of (4.877) made the most contribution to the model in predicting mothers’ use of skilled delivery care in the North East Region. NHIS made the less contribution to the model with Wald value of (0.065). Only religion with *p*-value of (0–026) and marital status with *p*-value of (0.012) were the only significant determinants or predictors of mothers’ use of delivery services. These recorded *p*-values < 0.05. This means the others variables were not significant predictors of mothers’ use of delivery care services. The odds ratio (Exp(B)) on this model revealed that educated mothers were three times (3.390) more likely than uneducated mothers to use delivery care. In the North East Region, Christians were 90% more likely than non-Christians to use delivery care.

According to the p-values column in Table 8, the only significant determinants or predictors of mothers' use of postnatal care services in the North East Region were marital status (*p*-value = 0.003) and religion (*p*-value = 0.012). The *p*-values for these two socio-demographic factors were less than 0.05.

From the data presented in Table 8; the model on mothers’ socio-demographic factors and use of postnatal care, the Exp(B) represents the ratio-change in the odds of mothers’ use of postnatal care for a one-unit change in the predictors (mothers’ socio-demographic). The Exp(B) for availability of skilled delivery personnel is equal to 1.028, which means that the odds of a mothers who had available skilled delivery personnel at the North East Region who utilised postnatal care were 1.028 times the odds of mothers without availability of skilled delivery personnel in the district. In other words, mothers who with availability of skilled delivery personnel were 1.028 times more likely to use postnatal care compared to mothers lack skilled delivery personnel. This study results implied that availability of skilled delivery personnel increases mothers’ use of postnatal by 1.028 times in the region. This means that more mothers were able to utilise postnatal care services following availability of skilled delivery personnel. Educated mothers were 0.941 (94%) more likely to use postnatal care services compared to uneducated women in the North East Region. Compared to non-Christians’ mothers, Christians’ mothers were 0.98 more likely to use postnatal care. This showed that the traditional beliefs made most mothers to fail to use postnatal care services.

The odds ratio, represents the change in odds of being in one of the outcome categories when the value of a predictor increases by one unit. Table 6 showed that, when compared to uneducated women, educated women were more likely to use ANC in the North East Region.

## Discussion

The findings revealed that the majority of the women in the study area gave birth at home due to factors such as distance, a lack of health care, and poverty. This adds to the labour complications during delivery. According to the literature, the location of a woman's delivery during childbirth is critical in reducing complications [27]. The findings proposed that primary healthcare facilities be established in rural communities in order to save the lives of rural women. Mothers should also be encouraged to give birth in a health care facility (hospital/clinic) in the presence of a trained professional health care practitioner such as a midwife, doctor, or nurse (Alkafaji and Al-Shamery, [28]). However, due to a lack of health facilities and qualified trained paramedic staff, the communities studied in Ghana lack professionals capable of providing maternal health care delivery services to mothers during childbirth. The findings contradict the literature, which claims that Ghanaian women meet the WHO maternal healthcare requirement by using facilities [2]. Furthermore, the findings revealed that most rural areas in Ghana lack health care centres and trained health care professionals required to provide rural mothers with delivery care, and the findings are consistent with the findings that most rural communities in Ghana lack healthcare facilities and healthcare professionals (Alkafaji & Al-Shamery, [28]). 

According to the findings, the most common reason given by mothers for not attending ANC during the first month of their pregnancy was that most women are unable to detect pregnancy during the first month. The timing of a mother’s access to ANC services is critical during pregnancy. The findings imply that pregnant women in the study area lack knowledge, which could be attributed to a lack of health education in rural communities. According to WHO [29] and Ghana Health Services [30], the first, second, and third months of pregnancy are the most critical for mothers to use ANC because health professionals can examine the mothers and the foetus and make necessary medical recommendations. The findings, however, contradict the WHO recommendation for the first, second, and third months of pregnancy. Furthermore, the findings contradict the Ghana Demographic and Health Survey (GDHS, [31]), which recommends monthly antenatal visits for the first seven months of pregnancy, after which pregnant women must visit every two weeks for the next eight months (GDHS, [32]). Mothers should seek ANC as early as the first or second month of their pregnancy. According to the findings, women should be encouraged to begin visiting the health centre during their previous pregnancy.

According to the findings, women usually discover they are pregnant during the second and third month after missing their menstrual period. This implied that most mothers, regardless of educational level, recognise the value of ANC. Surprisingly, some mothers were unable to recall the month in which they first visited ANC during their pregnancy. These mothers couldn’t tell you how many times they were admitted to the hospital during their previous pregnancy. This implied that some mothers do not see the need for ANC during pregnancy and, as a result, place less emphasis on the number of visits to the hospital for ANC. The findings contradict the literature, which suggests that maternal educational level has a significant influence on healthcare utilisation [6, 33]. 

According to the study’s findings, some mothers in the North East Region have had stillbirths in the last five years. This meant that some children died during delivery in the study area, which is concerning for the mothers who live there. Mothers who had no idea how many times they had attended ANC and those who did not visit ANC services in the early months of their pregnancy may have been among the few respondents who had stillbirths. The findings contradict previous research that found that family planning and fertility preference, as well as free maternal healthcare, reduced maternal and stillbirth rates in Ghana [34],[35] [36, 37].

According to the findings, socio-demographic factors such as women’s marital status, age, education, religious background, and distance have a significant relationship with mothers' use of health-care delivery services. The findings corroborated Dalaba et al. [8] and Dotse-Gborgbortsi et al.’s [12] findings that married women received assistance from their husbands during delivery to the nearest health facility. According to Banchani and Tenkorang [38], as well as Bellerose et al. [39], studies conducted in rural Africa revealed that husbands’ religious backgrounds influenced mothers’ use of skill delivery. The findings also lend support to the Andersen behavioural model, which enables characteristics on access to healthcare facilities.

According to the Chi-square test results, mothers’ marital status, age, education, religious affiliation, and distance have a significant relationship with mothers' use of maternal health care services during delivery in the North East Region. According to the literature, socio-demographic characteristics and behavioural variables have a significant influence on healthcare facility utilisation [4, 5, 9, 40].

The findings support the Andersen behavioural model, which states that characteristics of pregnancy age influence maternal service utilisation. The findings, however, contradict the literature, which indicates that married and educated women use antennal care services more than unmarried and uneducated women [16, 17, 41].

The problem here is that most mothers do not see the need to visit a health care facility after giving birth. Only married women and those whose religious beliefs support orthodox medicine visit the health care centre after delivery. The findings are consistent with previous research showing that women who receive antenatal and delivery care are more likely to receive postnatal care after childbirth [4, 5, 11, 42].

According to the study, the majority of mothers do not seek medical attention after giving birth. After giving birth, few mothers visited health care centres or used postnatal care services unless their child was sick. This could be due to the fact that the North East Region has many traditional healers who can administer herbal medicine to mothers and their children after birth, and thus mothers did not consider the need for postnatal care. The findings contradict the literature, which claims that free maternal health care has increased utilisation [36, 43],GHS, [30]).

The findings of this study confirmed the findings of the Ghana Demographic and Health Survey [31] and Ghana Demographic and Health Survey [44] studies, which found that a woman’s education was important in ANC utilisation in Ghana. Also, Vizheh et al. [14] discovered, through an analysis of DHS data from Bangladesh, Malawi, Bolivia, and the Philippines, that a woman’s education was a key determinant of mothers’ use of ANC services, just as Stanton et al. [24] did in their review of DHS data from developing countries. The North East Region's high percentage of educated mothers using ANC services calls for girl-child education.

The study also discovered that young mothers (those under the age of 30) were 40% less likely to use ANC services than older mothers. This supported the findings of Vasconcelos et al. [45]. In Bangladesh, women’s age was found to have a significant influence on their use of ANC. In addition, mothers with NHIS cards were more likely to use ANC than those without. This meant that the implementation of NHIS had had a significant impact on mothers' use of ANC services in the North East Region. The study also discovered that Christians were more likely than non-Christians in the North East Region to use ANC. According to the study findings, non-Christians seek advice from traditional healers and traditional birth attendants during pregnancy. This study confirmed the findings of Sunguya et al. [46], who found that only 32.7% of traditional believers visited ANC in Uganda in 2020. The study found that the people of the North East Region’s traditions influence their use of ANC. 

The study confirmed the findings of Saaka et al. [47], Nachega et al. [48], and Misu and Alam [49], which found that educated women were more likely than uneducated women to use delivery care. Most educated women emphasise the importance of delivery care and the importance for mothers to deliver at a centre under the supervision of skilled trained health professionals to reduce the risks during childbirth and the potential consequences that may occur during delivery. In addition, Anwar et al. [42] discovered that religious believers have a significant influence on maternal health care utilisation in Bangladesh. Similarly, Anselmi et al. [13] and Adamu [23] confirmed this by indicating that religious beliefs in developing countries such as Africa made achieving the SDGs of improved maternal health difficult. The findings of this study confirmed the findings of Bolduc [50] and Esena and Sappor [51], who found that marital status significantly predicts women's use of postnatal care services. The findings are consistent with the literature that the availability of skilled delivery personnel influence the health seeking behaviour of mothers (Adong et al., [4],Ghana Health Servivr Human Resource Annual Report, [52] and 2018; [53]).

The findings of the study demonstrated a theoretical relationship between the availability of skilled delivery paramedical staff, knowledge of pregnancy age, access, distance, and enabling characteristics such as poverty, gender, and traditional treatment, and the use of maternal healthcare services in rural communities. The study discovers a new relationship between the availability of skilled delivery professionals and access and distance, which has implications for the development of public health theories and models.

### The study implications and recommendations

According to the findings of the study, the following policy prescriptions should be implemented: increase access to skilled delivery care in the North East Region, as the study discovered that a lack of skilled delivery care limits mothers' access and use of MHCS. To supplement the availability of traditional healers and traditional birth attendants in the area, people should have access to health centres and professional health personnel. Furthermore, the Ghana Health Service should increase public health education in rural areas.

The findings strongly demonstrate that the Ministry of Health and the Ghana Health Service must take practical steps to increase education about the importance of having ANC with a skilled provider, as well as having the first 24 h of recommended primary healthcare services. The findings also show that education level, wealth status, marital status, antenatal care utilisation, health insurance coverage, getting medical help for self-getting permission to go, and distance to health facility all have a significant relationship with MCH utilisation. This implies that tailored educational and pro-MCH interventions must be streamlined in order to recognise the tones influenced by these factors.

The study recommended that mothers be educated about the importance of using skilled delivery care once more. By increasing MHS utilisation, women should be educated on the importance of maternal and child health. Women must regain economic empowerment in order to pay their own MHCS bills. Unemployed mothers should look for work, whether through trade, farming, or raising farm animals, to enable them to earn a living and pay their own medical bills while receiving MHS and they must not rely solely on their partners.

The study also recommended that the government organise training services for the area’s traditional birth attendants (TBAs) in order to provide them with the professional skills required to handle child delivery. The government should create a framework for regulating the activities of traditional herbal practitioners. They should also be trained, and the government should, if possible, integrate traditional and orthodox medicine in the area’s public hospital/clinic.

According to the study, health personnel who accept postings to the North East Region should be given special motivation packages by the local people and district assembly in order to help increase the number of health personnel in the area and solve the problem of insufficient health personnel in the area. The nurse and other health workers in the area educated mothers about the importance of receiving postnatal care after giving birth, as most did not appear to be doing so. To encourage others to learn from the few mothers who used all of these MHCS, the government should implement policies that reward mothers who use ANC, skilled delivery care, and postnatal care.

### Limitations of the study

There is no research without limitations (Prince & Murnan, [54]). Survivor bias may occur as this sample may not be the representative of the entire population due to the exclusion of individuals who did not survive or participate until the end of the study. To Ensure that the study’s data collection period was sufficiently long to capture a diverse range of participants, reducing the impact of survivor bias. Also, inclusion criteria to avoid unintentional exclusions. Another limitation was selection bias which may occurred in certain groups of the population and were more or less likely to be included in the study, leading to a non-representative sample. The simple random sampling was employed to minimise selection bias. Sampling bias was another potential limitation as selected sample may not accurately represent the entire population. Employing random sampling techniques to ensure that each member of the population has an equal chance of being selected. The study's findings may have limited generalisability if the sample did not represent entire population. The authors clearly defined the population of interest and acknowledge any limitations in generalisability. While these mitigation strategies can reduce the impact of biases, it's essential to acknowledge that this study is not entirely free from these limitations (Prince & Murnan, [54]).

## Conclusion

The study has contributed to a better understanding of the factors that influence mothers’ access to and utilisation of skilled delivery care services in rural communities throughout Ghana, particularly in the North East Region. According to the findings of the study, mothers' socio-demographic factors such as married status, age, education, religious background, and distance to health centre, as well as the availability of skilled delivery personnel, have a significant relationship with their access to and use of maternal care and skilled delivery care services. The study’s findings on access to skilled delivery care revealed that women’s marital status, age, education, religious background, and distance have a significant relationship with their use of skilled delivery care at health care facilities. Finally, the Chi-square (2) test results for postnatal care revealed that only mothers’ marital status and religious background were found to have a significant relationship with mothers’ use of postnatal care. Finally, in the North East Region, the only significant determinants or predictors of mothers' use of postnatal care services were marital status (*p*-value = 0.003) and religion (*p*-value = 0.012). 

### Ethical statement and informed consent

The study was ethically approved by the Committee on Human Rights, Publication, and Ethics College of Health Sciences, School of Medicine and Dentistry, Kwame Nkrumah University of Science and Technology. All participants signed/thumb printed written informed consent forms.
